# Static and Dynamic Performances of Chopped Carbon-Fiber-Reinforced Mortar and Concrete Incorporated with Disparate Lengths

**DOI:** 10.3390/ma14040972

**Published:** 2021-02-18

**Authors:** Yeou-Fong Li, Kun-Fang Lee, Gobinathan Kadagathur Ramanathan, Ta-Wui Cheng, Chih-Hong Huang, Ying-Kuan Tsai

**Affiliations:** 1 Department of Civil Engineering, National Taipei University of Technology, 1, Sec. 3, Chung-Hsiao E. Rd., Taipei 10608, Taiwan; x41012@gmail.com (K.-F.L.); gobiram0017@gmail.com (G.K.R.); 2 Institute of Mineral Resources Engineering, National Taipei University of Technology, Taipei 10608, Taiwan; twcheng@ntut.edu.tw; 3 Department of Architecture, National Taipei University of Technology, Taipei 10608, Taiwan; 4 Department of Environmental Information and Engineering, Chung Cheng Institute of Technology, National Defense University, P.O. Box 90047-82, Dasi, Taoyuan 33550, Taiwan; jeremytsai0406@gmail.com

**Keywords:** impact energy, compressive strength, flexural strength, chopped carbon fiber, mortar, concrete, blast wave

## Abstract

The impact load, such as seismic and shock wave, sometimes causes severe damage to the reinforced concrete structures. This study utilized different lengths of chopped carbon fibers to develop a carbon-fiber-reinforced mortar (CFRM) and carbon-fiber-reinforced concrete (CFRC) with high impact and anti-shockwave resistance. The different lengths (6, 12, and 24 mm) of chopped carbon fibers were pneumatically dispersed and uniformly mixed into the cement with a 1% weight proportion. Then the CFRM and CFRC specimens were made for static and dynamic tests. The compressive and flexural strengths of the specimens were determined by using the standard ASTM C39/C 39M and ASTM C 293-02, respectively. Meanwhile, a free-fall impact test was done according to ACI 544.2R-89, which was used to test the impact resistances of the specimens under different impact energies. The CFRM and CFRC with a length of 6 mm exhibit maximum compressive strength. Both flexural and free-fall impact test results show that the 24 mm CFRM and CFRC enhances their maximum flexural strength and impact numbers more than the other lengths of CFRM, CFRC, and the benchmark specimens. After impact tests, the failure specimens were observed in a high-resolution optical microscope, to identify whether the failure mode is slippage or rupture of the carbon fiber. Finally, a blast wave explosion test was conducted to verify that the blast wave resistance of the 24 mm CFRC specimen was better than the 12 mm CFRC and benchmark specimens.

## 1. Introduction

Reinforced concrete structures are sometimes subjected to seismic and high impact loadings, which might cause catastrophic damage. An impact load might be an aircraft taking off or landing on a runway, or a heavy vehicle passing over a bridge expansion joint; in a harbor, a ship may collide with a wharf due to the movement of the waves. These impact loadings cause damage, such as cracks and spalling, to the reinforced concrete pavement. Generally, natural and manmade fibers incorporated into reinforced concrete structures can improve the durability and toughness and can reduce the shrinkage of concrete. Recently, carbon-fiber-reinforced mortar (CFRM) and carbon-fiber-reinforced concrete (CFRC) are usually being used in the repair, rehabilitation, and rebuilding work of civil engineering infrastructures.

Fiber-reinforced mortar (FRM) enhances the tensile and impact resistance, and fiber is used as reinforcement to increase its strength. Concrete is a brittle material and prone to cracks or damage when subjected to an external force, and the load-bearing capacity and its serviceability will be reduced. If the compression and impact resistance of concrete structures can be improved, then the service life of the various reinforced concrete structures can be prolonged. Fiber-reinforced concrete (FRC) has good mechanical properties and is used in multiple construction environments.

In the last few decades, many studies have been conducted on the performance of different fibers applied in mortar and concrete. Concrete reinforced by steel rebars and polypropylene fiber has shown an increase in mechanical performance on flexural strength and improved ability to impact resistance [1]. Fiber-reinforced lightweight foamed concrete integrated with glass-fiber-reinforced plastics (GFRP) mesh was placed in the tensile region; the polymer fibers increased the flexural capacity of the beams, especially for the low-density specimens and for the higher contents of fibers [2]. Glass-fiber-reinforced concrete with 1% volume fraction glass fiber can enhance flexural strength, and the impact resistance and mechanical properties are strengthened by different lengths of fiber and volume content [3,4,5]. The various volume fraction and the water–cement ratios of silica fume incorporated with steel fibers can improve the ductility and impact resistance. It was also found that the impact resistance of reinforced concrete increased when silica fume and fine aggregate were replaced by cement and rubber fiber. The fiber-reinforced concrete proved that the energy absorption capacity of polypropylene was superior to that of cellulose [6,7].

Several studies reveal the effect of adding different fibers on the mechanical properties of fiber-reinforced concrete: The impact resistance of self-consolidating rubberized concrete (SCRC) can be determined by the reinforced steel fiber and synthetic semi-rigid fiber; the addition of synthetic semi-rigid fiber to self-filling rubber concrete can improve impact resistance. Geopolymer concrete reinforced with mono fiber has a remarkable enhancement in its impact strength and fracture toughness. It was concluded that the steel fiber–polypropylene fiber reinforced specimen has the best toughness, with a proportion of 1.5%, and it takes repeated impacts to reach complete failure [8,9,10,11].

Carbon fiber is a lightweight composite material, which does not corrode, degrade, or fatigue. Due to its high specific strength, it is used in the aerospace industry, for automotive parts and sports equipment, and in civil engineering. The content and length of carbon fiber in concrete affect performance on the strength of various aspects. As the amount and length of additive carbon fiber increases, the impact resistance significantly improves. The failure of fiber-reinforced concrete paste is mainly due to the force endured beyond the bonded force between surfaces [12,13,14,15,16,17]. The cement composites with uniformly distributed carbon fiber provide high efficiency, which was identified by an electron microscope. In addition, fiber-dispersion processes were examined with various studies in the cement composites [18,19,20]. The chopped carbon fiber was treated for the removal of residual silane by using chemical and physical methods, and it was detected by GC/MS (Gas Chromatography/Mass Spectrometry) testing. The physical treatment exhibits high compressive strength of 14.1%, as compared with chemical treatment [21]. The chopped fiber is a lightweight material, and its mechanical behavior is enhanced when mixed into concrete. The results show that the fiber is uniformly distributed, stress acts in a different direction, and the chopped fiber improves the composite material performance from the brittle failure [22]. The multi-stage procedure was carried out with fiber contour modelling techniques and subsequent hierarchical multiscale analysis [23]. Ultrasonic waves and vibration were used to improve the dispersion of carbon fiber in cement composites [24]. The effect of the microstructure of CFRC on their macrostructure and the mechanical properties of CFRC specimens were investigated [25]. The strength of the fiber-reinforced lightweight aggregate concrete increases with different fiber volume fractions embedded in the cementitious matrix [26,27,28,29].

Chopped carbon fiber was used in this study, to determine the static (compression and bending) and impact behavior of concrete and mortar with different lengths of fiber (6, 12, and 24 mm). The carbon fibers were separated by dispersion and then incorporated into the cement with a 1% weight proportion. A pneumatic process was additionally used to disperse the carbon fiber for uniform distribution in the concrete and mortar.

## 2. Materials

The carbon fibers were chopped at different lengths (6, 12, and 24 mm), which were uniformly mixed in slurry forms for mortar and concrete. In this section, the materials, experimental methods, and equipment are listed; they include various material properties of carbon fiber, aggregates, and cement. Carbon-fiber tensile strength and elastic modulus are superior to other fibers; the properties are listed in Table 1, respectively. The data were obtained from the manufacturers and journal papers [11,30,31]. The carbon-fiber weight proportions were precise by 0.5%, 1%, and 1.5% in the flexural test, which was verified with a 0.45 water–cement ratio of mortar and concrete. Among those, the addition of 0.5% carbon fiber shows that the weight proportion is less effective with the mortar and concrete, and it does not increase the strength significantly. The strength increases with the addition of 1.5% carbon fiber, but the specimens are too dry to compact in the model, which shows many voids and a honeycomb in the concrete. Finally, the addition of 1% carbon fiber indicates that the strength is almost similar to 1.5%, and it is used in this study. Static and impact load experimental tests determined fiber resistance in the mortar and concrete.

### 2.1. Cement

Portland cement provides overall strength, and it is used within one month after the manufacture date. Note that the cement should not be in direct contact with the ground, in order to avoid moisture absorption. The carbon fiber is included in a cement matrix, in a dry state composition.

### 2.2. Carbon Fiber

**Figure 1:**
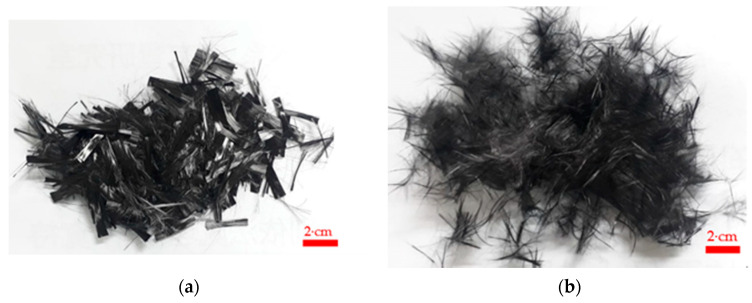
Dispersion process and appearance of fiber: (**a**) chopped carbon fiber and (**b**) chopped carbon fiber after pneumatic separation.

Carbon fiber applications are exploited in the aerospace industry, automobile industry, and other fields, which have high specific strength and fatigue resistance. The short carbon fiber was acquired from Tairylan Division, Formosa Plastics Group, Taipei, Taiwan, R.O.C. [30], and the properties are listed in Table 2. PAN-based carbon fiber was included in the cement mixture, by the dispersion of linear strands; the fibers must be dispersed before mixing, to ensure that the fibers were evenly mixed into the cement base material. The chopped carbon fiber is caked during mixing because it has relatively high slenderness. The chopped carbon fiber’s appearance and pneumatic dispersion are shown in Figure 1.

**Figure 2:**
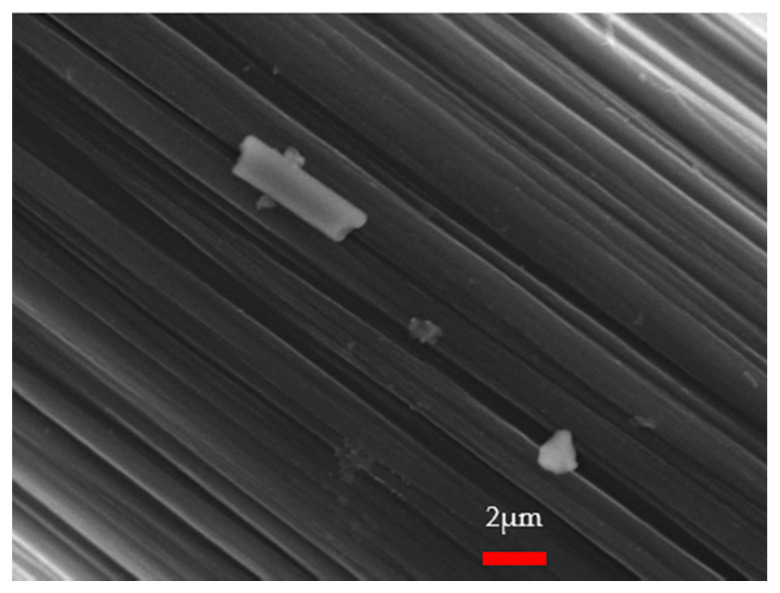
SEM observation on the surface of the chopped carbon fiber.

The carbon fiber surface microscopy was examined with a scanning electron microscope (model: JSM-7610F, JEOL, Tokyo, Japan), at the Department of Molecular Science and Engineer Lab, National Taipei University of Technology. The carbon-fiber-surface SEM observation is shown in Figure 2, with a highly magnified image. The presence of silane on the surface of carbon fiber was observed in GC/MS testing [21], as shown in Figure 2. The silane on the surface of the chopped carbon fiber might interfere with the bonding strength between the carbon fiber and cement.

### 2.3. Carbon-Fiber-Reinforced Mortar (CFRM)

Fiber-reinforced mortar (FRM) presents high flexural deformation; it can increase durability, reduce shrinkage, and improve toughness. The carbon fiber can restrain mortar cracking and prevent fracture failure; it can also offer improvement to the mechanical and physical properties of mortar. In this study, the water–cement ratio of 0.4 was used in the CFRM specimens, and sand was 105% of the cement weight. The dispersed carbon fiber was mixed in the dry stage and then wet state, to aid in the uniform distribution in the mortar.

### 2.4. Carbon-Fiber-Reinforced Concrete (CFRC)

Generally, fibers are usually used in concrete, to control cracking due to plastic shrinkage and drying shrinkage. Some types of fibers could produce greater impact resistance, abrasion resistance, and shatter resistance in concrete. Since the modulus of elasticity of carbon fiber is higher than it is for the plain concrete, it can help the CFRC carry the load by increasing the tensile strength. To ensure that each fiber strand is effective, it is necessary to disperse the fibers uniformly in the concrete. In this study, the water–cement ratio of the CFRC was 0.45, and the sand weight was the same as the CFRM. As the component of a composite material that resists compressive stress, the aggregate was 225% of the cement weight. The fineness modulus of aggregates for CFRC specimen was 6.01, as shown in Table 3.

## 3. Experimental Methods and Setups

A series of tests were conducted according to ASTM and ACI standards [32,33,34], to investigate the effect of different lengths of carbon fibers (6, 12, and 24 mm) on the compressive strength, flexural strength, and impact energy of the CFRM and CFRC. The CFRC and the CFRM specimens were cured at 28 days. The CFRM and CFRC were tested by using a universal testing machine and free-fall impact equipment. The compressive and bending tests were conducted by using a universal testing machine (HT-9501 Series. Hong-Ta, Taipei, Taiwan), with a load cell (WF 17120, Wykeham Farrance, Milan, Italy), at the Department of Civil Engineering, National Taipei University of Technology. The experimental setup and the process of compression, bending, and impact tests are listed below.

### 3.1. Compressive Test

**Figure 3:**
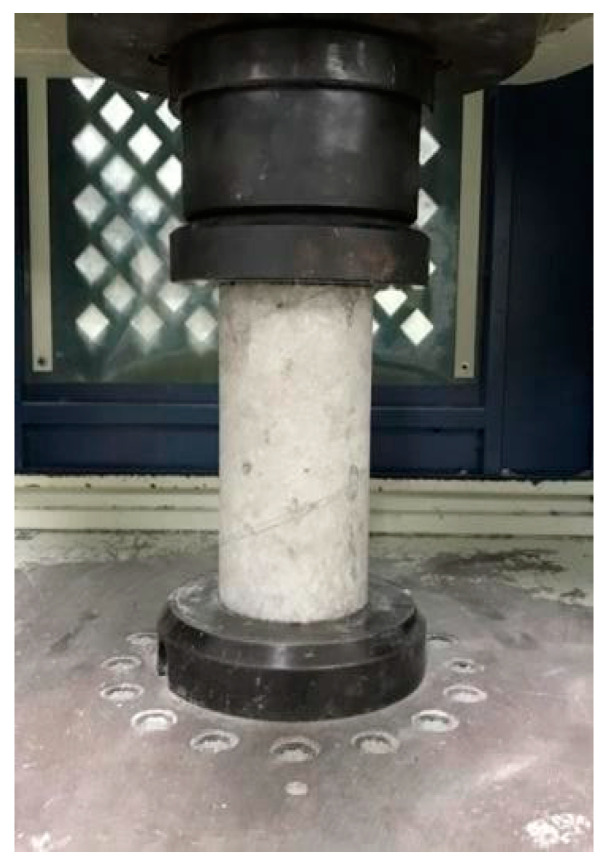
Compression test of carbon-fiber-reinforced concrete (CFRC) cylindrical specimen.

The carbon-fiber-reinforced composites were tested under ASTM C39/C 39M-01 standards [32]. The cylindrical specimen was placed in a universal testing machine with a loading rate of 900–1800 N/s (strain rate of 10^−6^/s to 10^−4^/s), which was acted on the flat surface of the specimen, and the dimension was ⌀10 cm × 20 cm [32], respectively. The cylindrical specimen was tested under the material laboratory of the Department of Civil Engineering, National Taipei University of Technology, Taiwan. Figure 3 shows the compression test experimental fixture of CFRC.

### 3.2. Three-Point Bending Test

**Figure 4:**
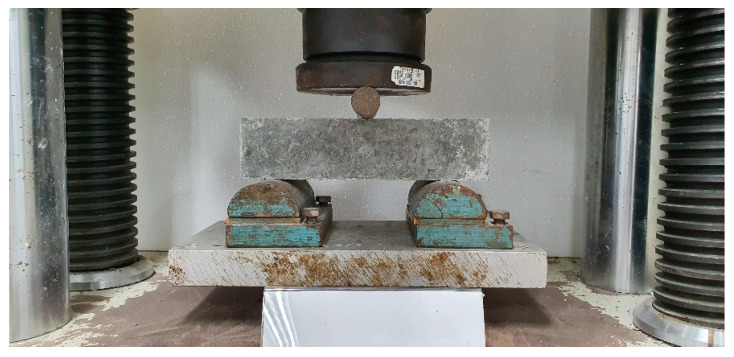
Three-point bending test of CFRC setups.

According to ASTM C 293-02 [33], the CFRM and CFRC specimens were tested with dimensions 28 cm × 7 cm × 7 cm [33]. The chopped carbon fibers were distributed uniformly in mortar and concrete, after using the pneumatic method. The CFRM and CRFC were tested under a loading rate of 1.2 MPa/min, respectively. Figure 4 shows the setup of the three-point bending test; the specimen’s flexural strength is determined in the universal testing machine.

### 3.3. Free-Fall Impact Test

**Figure 5:**
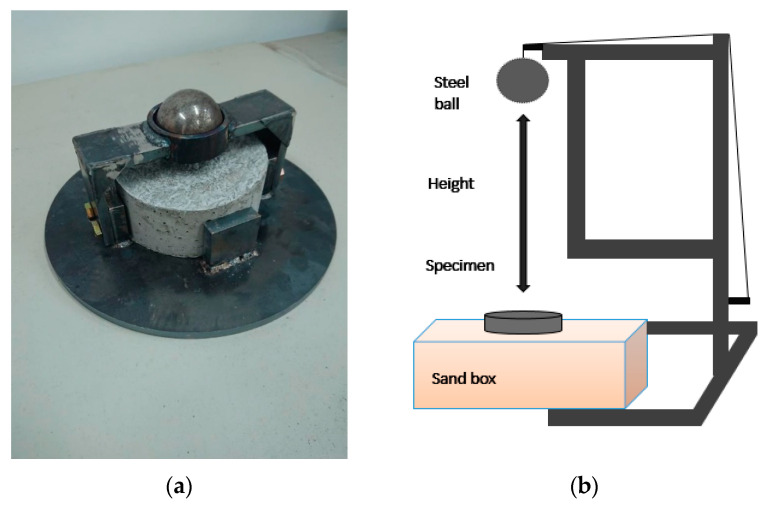
Free-fall impact test: (**a**) fiber-reinforced concrete with test equipment and (**b**) free-fall impact test setup.

The free-fall impact tests of CFRM and CFRC were conducted under ACI 544-2R [34]. The sieving dispersion was involved in the specimen preparation process. The given length and quantity of carbon fibers were mixed evenly in the dry cement. The dry-mixing process comprised the period of proper time, followed by wet mixing after adding the aggregates and water. The concrete and mortar cylindrical specimen’s dimensions were ⌀150 mm in diameter and 64 mm in thickness, and the specimens were placed in the sandbox, for impact-load performance. The specimens were tested with different impact energies, in the impact test, by using an iron ball at the heights of 100 to 500 cm. Figure 5a shows the standard drop-weight-test cylindrical specimens and equipment, and the free-fall impact test setup is shown in Figure 5b.

The CFRM and CFRC were tested with different impact energies; the string was used to hang the mass steel ball at a certain height. The CFRM and CFRC specimens’ surfaces resist a single impact at high energy and repeated impact at lower energy. The impact energy can be represented as follows.

*E* = *m* × *g* × *h* (1)

In Equation (1), *E* is potential energy (J), *m* is mass (kg), *g* is gravity acceleration (m/s^2^), and *h* is height (m). The CFRC and CFRM are examined at different energies with a single and repeated free-fall impact test. The CFRC and CFRM with different lengths of fiber (6, 12, and 24 mm) possess better mechanical performance than the benchmark.

## 4. Results and Discussions

The disparate lengths of fiber 6, 12, and 24 mm were incorporated with cement, to prepare CFRM and CFRC specimens. The test results of compressive, bending, and impact performance were obtained with the different fiber lengths of carbon fiber in CFRM and CFRC specimens.

### 4.1. Compressive Test Results

In this subsection, the CFRM and CFRC compressive strengths were tested with different lengths of fiber and then compared with a benchmark specimen (without added carbon fiber). Each test group had three specimens.

#### 4.1.1. Compressive Test Results of CFRM

Table 4 shows that the specimen name C-B-M represents the benchmark mortar specimen without carbon fiber; C-L6-M, C-L12-M, and C-L24-M represent CFRM adding 6, 12, and 24 mm chopped carbon fiber. Table 4 shows the benchmark specimen’s compressive strength and CFRM with a disparate length of carbon fiber. The chopped carbon fibers using the pneumatic dispersion process facilitated a uniform distribution in the cement and contributed to enhancing the CFRM overall strength. The 6 mm chopped carbon fiber increases the compressive strength by 22.2%, on average (39.75 MPa), compared with the benchmark specimen, while 12 and 24 mm enhanced it by 14% and 11.3%, on average.

The strength of the CFRM is higher than that of the benchmark mortar specimen, and increasing compressive strength is dependent on the length of the carbon fiber. The chopped 6 mm carbon fibers were uniformly inhabited in the cylindrical specimens because the length of 6 mm fibers is shorter than the others, and it occupies more volume than the longer fiber in the concrete matrix with the same weight proportion. The compressive strength of CFRM specimen was increased by reducing the length of the carbon fiber.

#### 4.1.2. Compressive Test Results of CFRC

In Table 5, the specimen name C-B-C represents the benchmark concrete specimen without carbon fiber; C-L6-C, C-L12-C, and C-L24-C represent CFRC added with 6, 12, and 24 mm lengths of chopped carbon fibers. Table 5 shows the compressive strength of the benchmark specimen and CFRC specimens with different carbon fiber lengths. The 6 mm length of CFRC increases resistances by 25.3%, on average, compared to the benchmark concrete. The 12 and 24 mm carbon fiber lengths increase the compressive strength by 14.4% and 4.4%, on average, respectively.

The C-L6-C chopped carbon-fiber-reinforced concrete has a maximum compressive strength than the benchmark and other fiber-reinforced concrete (such as C-L24-C and C-L12-C). Because the length of 6 mm fibers is shorter than the others, they possess more volume than the longer fibers in the concrete matrix with the same weight proportion; that is, 6 mm fiber occupies two times more space than the 12 mm carbon fibers, and four times more space than the 24 mm carbon fibers.

### 4.2. Three-Point Bending Test results

The chopped CFRM and CFRC increase their flexural strength, compared to the standard benchmark specimens. In this subsection, the flexural strength of CFRM and CFRC with different lengths of fiber are compared with the benchmark specimens (without added carbon fiber). The flexural strength of fiber-reinforced mortar and concrete is discussed below. Each test group has three specimens.

#### 4.2.1. Three-Point Bending Test Results of CFRM

As shown in Table 6, the 24 mm chopped CFRM (F-L24-M) increases its flexural strength up to 42.14% more than the flexural strength of the benchmark specimen. Similarly, the flexural strength of CFRM with 6 and 12 mm lengths of carbon fibers has higher strength than the benchmark specimen (24.57% and 29.06%, respectively). The flexural strength of CFRM is increased with the increasing length of the fiber.

From the above test results, we can see that the length of the long carbon fiber (24 mm) enhances its maximum strength in the CFRM. This is because the longer carbon fiber can resist tensile force, and its failure mode is fracture failure instead of slip failure (see Section 4.4, “Optical Microscopic Observation”).

#### 4.2.2. Three-Point Bending Test Results of CFRC

Table 7 shows that the CFRC specimens with the length of 6, 12, and 24 mm carbon fibers increase their flexural strength, compared with the benchmark specimen (F-B-C), by 17.82%, 22.92%, and 27.07%, respectively. The flexural strength of CFRC is increased by increasing the length of the chopped carbon fiber.

As seen from Table 6 and Table 7, the average flexural strength of CFRC is less than the average flexural strength of the CFRM at the same length of chopped carbon fiber.

### 4.3. Free-Fall Impact Test Results

A steel ball was fixed at a given height and repeatedly impacted the top surface of the specimens, as shown in Figure 5b. The number of impacts under different impact energies was recorded. Each test group has five specimens.

#### 4.3.1. Free-Fall Impact Test of CFRM

The impact numbers of benchmark and CFRM specimens under different impact energies are shown in Table 8. It was observed that the longer carbon fiber could resist more impacts at lower impact energies. For instance, under 98 J impact energy, the maximum number of impacts at the failure of CFRM specimens with the length of 24, 12, and 6 mm carbon fiber were 245, 35, and 31, respectively. The 24 mm CFRM resists repeated impact and exhibits higher effectiveness; it also attained more than 2000 impact numbers under 49 J. The one-time impact failure energy of 24 mm carbon-fiber-reinforced concrete was 339 J, which is much larger than the benchmark mortar specimen.

Generally, the longer fiber has better impact resistance. Table 8 shows that the impact resistance is predicted under repeated loading with an impact energy of 98 and 49 J. However, the impact resistance under high impact energy is relatively difficult to assess. This is because the impactor was sometimes dropped on the surface of either the gravel or fiber during the test, causing the impact number to be unstable when the steel lump hits the specimens at high impact energy.

**Figure 6:**
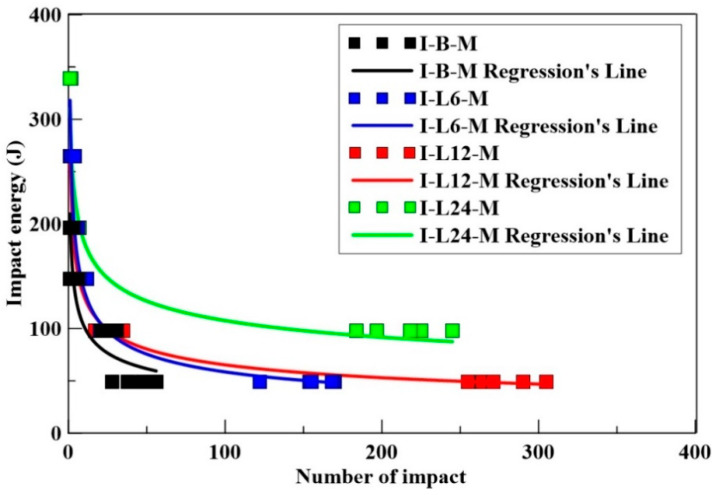
The impact energy/number relationship of benchmark and CFRM specimens.

The relationships of the number of impacts and impact energy of CFRM are shown in Figure 6. As seen from Figure 6, the impact resistance (impact energy and impact number at failure) of CFRM specimens was higher than that of the benchmark.

#### 4.3.2. Free-Fall Impact Test of CFRC

The impact numbers of benchmark and carbon-fiber-reinforced concrete specimens under different impact energies are shown in Table 9. From the impact test results, we can see that the CFRC specimen’s failure was not caused by the accumulation of energy. For instance, under 147 J impact energy, the maximum impact numbers at the failure of CFRC specimens with the length of 6, 12, and 24 mm carbon fibers were 4, 6, and 11, respectively. Moreover, under 49 J impact energy, the maximum impact numbers at the failure of CFRC specimens with the length of 6, 12, and 24 mm carbon fibers were 48, 153, and ≥2000, respectively. It is worth mentioning that, under 49 J impact energy, the 24 mm CFRC maximum number of impacts before failure was more than 2000 times. The 24 mm carbon fiber length in CFRC has better impact resistance at lower impact energies. As seen in Table 9, the impactor with high impact energy sometimes hits the surface of gravels or fibers. The increased impact resistance can be captured merely under the repeated loading of 98 and 49 J.

**Figure 7:**
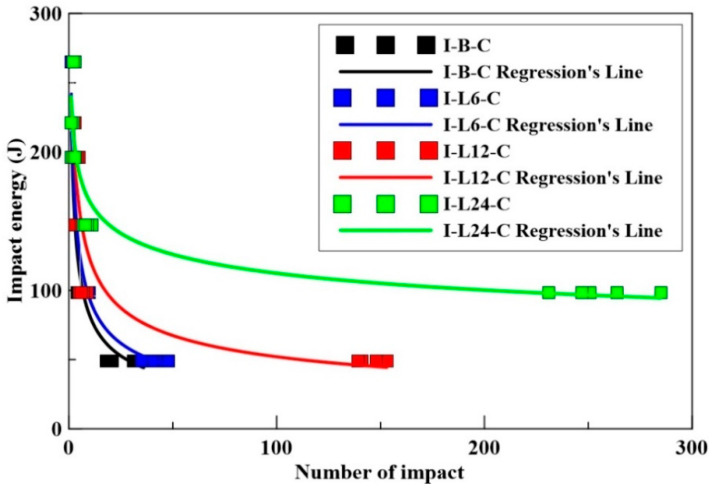
The impact energy/number relationship of benchmark CFRC specimens.

**Figure 8:**
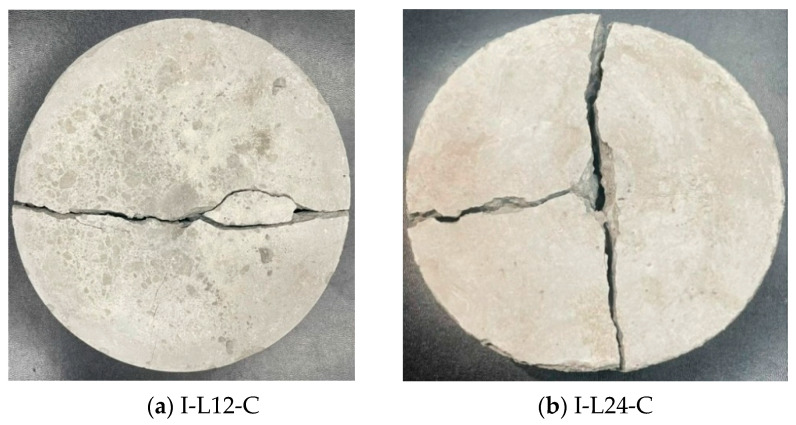
The failure photo of the specimen under repeated impact at 98 J: (**a**) I-L12-C and (**b**) I-L24-C.

The impact energy/number relationship of benchmark and CFRC specimens is shown in Figure 7. As seen in Figure 7, the CFRC with 24 mm carbon fiber (I-L24-C) specimen has a maximum impact energy resistance, as compared with the benchmark and other CFRC (I-L6-C, and I-L12-C). Figure 8 shows the failure image of specimen I-L12-C in two pieces and I-L24-C, in three pieces, under repeated impact.

### 4.4. Optical Microscopic Observation

**Figure 9:**
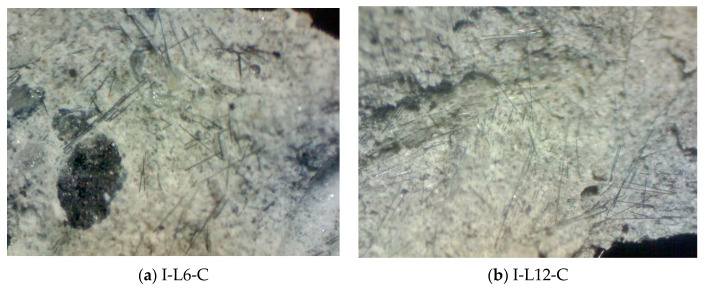

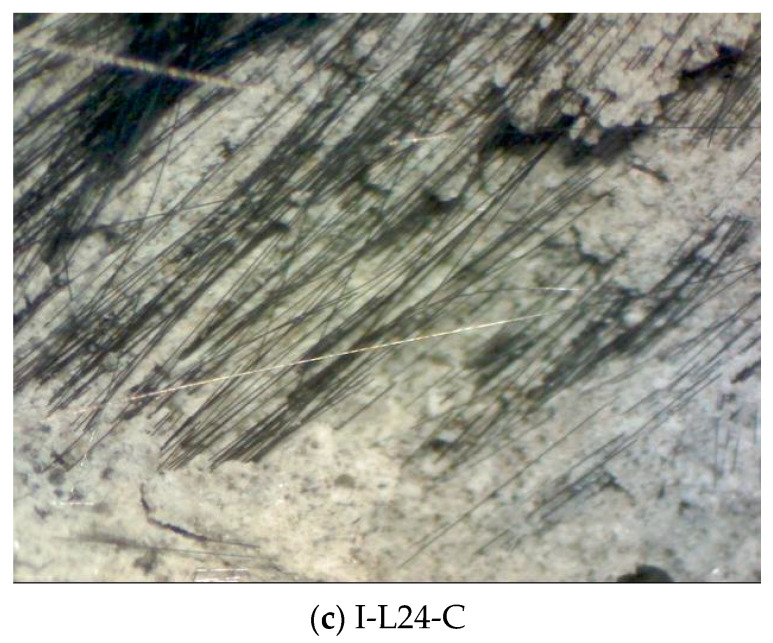
An optical microscopic image of the fiber-reinforced concrete surface after free-fall impact test: (**a**) I-L6-C, (**b**) I-L12-C, and (**c**) I-L24-C.

After the impact test, the failure surfaces of the CFRC specimens were analyzed by optical microscopy (model: MSH631-B, Hamlet, New Taipei City, Taiwan), with a high magnification range between 200 and 400, at the material laboratory of the Institute of Mineral Resources Engineering, National Taipei University of Technology. Figure 9 shows the photomicrograph of the fractured surface of the CFRC specimens. The CFRC surface shows that the fiber is well dispersed and uniformly distributed in the concrete, because each of the linear strands of fiber can be seen in the photo.

The CFRC fracture surface from the photomicrograph is obtained from an optical microscope, as shown in Figure 9, which shows that the I-L6-C specimen contribution with reinforced concrete is less effective than the I-L12-C and I-L24-C specimens. Because the fibers in I-L6-C mostly occur as slippage failure, while I-L12-C and I-L24-C had more rupture failure instead of slippage failure. The slippage and rupture failure occurred when the strength was increased with the increasing length of fiber in the impact-test specimen.

## 5. Blast Wave Explosion Test Verification

The blast wave explosion test was used to verify the anti-blast wave resistance of different carbon fiber lengths of CFRC. In Table 10, the specimen BW-B-C represents the benchmark specimen, which had no added carbon fiber. Specimen BW-L12-C and specimen BW-L24-C represent CFRC with 12 and 24 mm chopped carbon fiber added.

**Figure 10:**
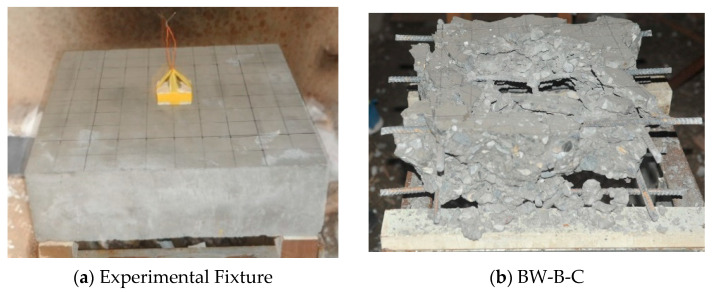

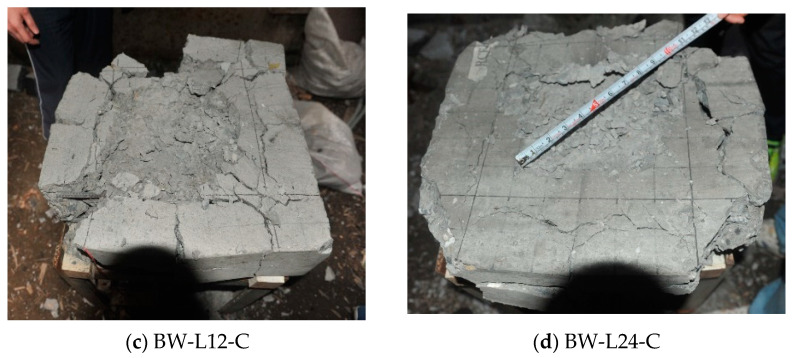
(**a**) Experimental fixture, (**b**) crushing surface of benchmark (BW-B-C), (**c**) spalling surface of BW-L12-C, and (**d**) spalling surface of BW-L24-C.

Figure 10a shows that the dynamite C4 (150 g) is placed on the top surface of a CFRC slab, to demonstrate the blast-wave resistance of chopped fiber reinforcement. Figure 10b shows the failure mode of an ordinary reinforced concrete slab specimen (without carbon fiber), the shock wave caused by the explosion, and the specimen completely crushed. Figure 10c shows specimen BW-L12-C after the explosion; an obvious crater can be observed on the slab’s top surface. Moreover, Figure 10d shows the post-test photograph of specimen BW-L24-C; minor spall damage is observed at the rear side of the slab, indicating tensile strength improvement.

Table 10 shows the failure mode, damage diameter, and depth of CFRC specimens after the blast wave explosion. The specimen BW-B-C shows breaching failure, and the specimens BW-L12-C and BW-L24-C both present spalling failure. The spalling depth of specimen BW-L12-C is 5 cm, and the inner/outer circle diameters of the spalling surface are 36 and 50 cm, respectively. Similarly, the spalling depth of specimen BW-L24-C is 4.3 cm, and the inner/outer circle diameters of the spalling surface are 31 and 35 cm. The blast-wave-explosion test result shows that the specimen BW-L24-C has better anti-blast performance than other specimens.

## 6. Conclusions

The chopped carbon fibers (6, 12, and 24 mm) enhanced the static and impact load performances of mortar and concrete. The compressive strength increased with a decrease in the length of chopped carbon fibers. The pneumatic dispersion process also aids in distributing the fiber uniformly. The 24 mm chopped carbon-fiber-reinforced cement composites better resist repeated impacts at low energy than the composites reinforced by other lengths of carbon fibers. The conclusions are as follows:

1. Among them, the C-L6-C specimen exhibits good compressive strength, which is 25.3% (40.28 Ma) more than the C-B-C specimen. The C-L12-C and C-L24-C specimens also increase the compressive strength by 14.4% and 4.4%, respectively. The inclusion of 6 mm carbon fiber exhibits the maximum compressive strength. Compared to the C-B-M specimen, the C-L6-M, C-L12-M, and C-L24-M specimens enhance their compressive strength by 22.2%, 14%, and 11.3%, respectively. The compressive-strength-enhancement effect decreases by increasing the length of the fiber.
2. The flexural strength of the F-L24-M specimen increases its strength, compared with the benchmark, by 42.14%. The CFRM specimens F-L6-M and F-L12-M also enhance their strength by 24.57% and 29.06% respectively. The CFRC specimens F-L24-C improve flexural strength by 27.07 MPa. The specimens F-L6-C (17.82%) and F-L12-C (22.92%) also increase their strength, compared with the benchmark (F-B-C), which shows that flexural strength increases as the length of the chopped carbon fibers increases.
3. The impact number of the CFRM specimen exhibits that the specimen I-L24-M can sustain high impact energy at 339 J, and specimen I-L24-M has maximum impact numbers, as compared with specimens I-L12-M and I-L6-M, at different impact energies. The I-L24-C specimen exhibits higher impact-energy resistance, as compared with the benchmark and other specimens, at different impact energies. The failure impact numbers of the CFRM and CFRC specimens show that the strength is increased by increasing the chopped-carbon-fiber length.
4. In the blast test, the specimen BW-B-C exhibits crushing failure, and the specimens BW-L12-C and BW-L24-C exhibit spalling failure. The spalling depth of specimen BW-L12-C is 5 cm, and the inner/outer circle diameters of the spalling surfaces are 36 and 50 cm, respectively. Similarly, the spalling depth of specimen BW-L24-C is 4.3 cm, and the inner/outer circle diameters of the spalling surfaces are 31 and 35 cm. The test result indicates that the specimen BW-L24-C has better anti-blast performance than other specimens.
5. The CFRC test results show that CFRC can be applied in the reinforced concrete structures of bridge expansion joints and aircraft runways, due to its excellent compressive, bending, and impact performances. The CFRC can be applied in the reinforced-concrete structures, to prevent damage from the blast wave and seismic loading.
6. The CFRM test results show that CFRM can be applied in the repair work in the reinforced concrete structure, due to its excellent mechanical performance.

## Figures and Tables

**Table 1:** The material properties of different fibers [11,30,31].

	Fiber	Carbon	E-Glass	Steel	Polypropylene
Material Property					
Density (g/cm^3^)		1.78	2.55	7.8	0.91
Tensile Strength (MPa)		4900	2750	1300	500
Specific Strength (MPa·cm^3^/g)		2750	1078	165	550
Elastic Modulus (GPa)		240	72	200	7

**Table 2:** Material properties of chopped carbon fiber [30].

Material Property	Value
Tensile strength (MPa)	4900
Tensile modulus (GPa)	250
Elongation (%)	2.0
Density $(g/{\mathrm{cm}}^{3}$)	1.78
Fiber diameter (μm)	7

**Table 3:** Fineness modulus of aggregates.

Sieve No.	Sieve Size (mm)	Weight Retained (g)	Percent Retained (%)	Cumulative Percent Retained (%)
3/2″	37.5	0	0	0
3/4″	19.0	672.3	23	23
3/8″	9.5	1352.4	46.2	69.2
No. 4	4.75	10.2	0.3	69.5
No. 8	2.36	165.6	5.7	75.2
No. 16	1.18	236.7	8.1	83.3
No. 30	0.60	178.2	6.1	89.4
No. 50	0.30	146.7	5	94.4
No. 100	0.15	83.7	2.9	97.3
Pan	–	79.2	2.7	100
Total	–	2925	–	Cumulative = 6.01

Fineness modulus (F.M.) = 6.01.

**Table 4:** Compressive strength of benchmark and different fiber lengths of carbon-fiber-reinforced mortar (CFRM) specimens (unit: MPa).

Specimen	C-B-M	C-L6-M	C-L12-M	C-L24-M
Compressive Strength	33.08	39.18	36.64	36.25
	33.45	40.83	38.27	36.70
	31.09	39.01	36.39	35.28
Average	32.54	39.75	37.10	36.22
Increase (%)	0	22.2	14	11.3

Note: C, compressive; B, benchmark; L6, 6 mm carbon fiber; L12, 12 mm carbon fiber; L24, 24 mm carbon fiber; M, mortar.

**Table 5:** Compressive strength of benchmark and different fiber lengths of CFRC specimens (unit: MPa).

Scheme 6	C-B-C	C-L6-C	C-L12-C	C-L24-C
Compressive Strength	32.87	39.42	37.62	34.47
	31.36	40.99	36.59	33.09
	32.16	40.46	36.14	33.09
Average	32.15	40.28	36.78	33.55
Increase (%)	0	25.3	14.4	4.4

Note: C, compressive; B, benchmark; L6, 6 mm carbon fiber; L12, 12 mm carbon fiber; L24, 24 mm carbon fiber; C, concrete.

**Table 6:** Flexural strength of benchmark and different fiber lengths CFRM specimens (unit: MPa).

Specimen	F-B-M	F-L6-M	F-L12-M	F-L24-M
Flexural Strength	7.26	9.58	10.29	10.68
	7.96	9.74	9.42	10.05
	7.49	8.98	9.60	11.54
Average	7.57	9.43	9.77	10.76
Increase (%)	–	24.57	29.06	42.14

Note: F, flexural; B, benchmark; L6, 6 mm carbon fiber; L12, 12 mm carbon fiber; L24, 24 mm carbon fiber; M, mortar.

**Table 7:** Flexural strength of benchmark and different fiber lengths of CFRC specimens (unit: MPa).

Specimen	F-B-C	F-L6-C	F-L12-C	F-L24-C
Flexural Strength	7.38	8.68	9.07	9.24
	7.78	8.64	9.09	9.64
	7.22	9.05	9.34	9.56
Average	7.46	8.79	9.17	9.48
Increase (%)	–	17.82	22.92	27.07

Note: F, flexural; B, benchmark; L6, 6 mm carbon fiber; L12, 12 mm carbon fiber; L24, 24 mm carbon fiber; C, concrete.

**Table 8:** Impact number of benchmark and CFRM specimens at failure, under different impact energies.

	Impact Energy (J)	339	264	196	147	98	49
Specimen							
I-B-M		–	–	1–3	4–6	22–31	38–56
I-L6-M		–	1–4	3–6	8–12	20–31	122–170
I-L12-M		–	1–3	2–5	3–4	17–35	255–305
I-L24-M		1–2	3–4	4–7	6–11	184–245	≥2000

Note: I, impact; B, benchmark; L6, 6 mm carbon fiber; L12, 12 mm carbon fiber; L24, 24 mm carbon fiber; M, mortar.

**Table 9:** Impact number of benchmark and CFRC specimens at failure, under different impact energies.

	Impact Energy (J)	264	220	196	147	98	49
Specimen							
I-B-C		–	1–2	1–3	3–4	4–7	18–36
I-L6-C		1	1–2	2–4	3–4	6–10	35–48
I-L12-C		2–3	2–3	3–5	3–6	24–30	139–153
I-L24-C		1–3	1–2	2–3	7–11	231–285	≥2000

Note: I, impact; B, benchmark; L6, 6 mm carbon fiber; L12, 12 mm carbon fiber; L24, 24 mm carbon fiber; C, concrete.

**Table 10:** Failure mode, damage diameter, and the depth of benchmark and CFRC specimens.

Specimen	Failure Mode	Inner/Outer Diameter (cm)	Depth (cm)
BW-B-C	Crushing	–	Penetrate
BW-L12-C	Spalling	36/50	5
BW-L24-C	Spalling	31/35	4.3

## Data Availability

Not applicable.
